# 增强剂量免疫化疗联合一线自体外周血造血干细胞移植治疗年轻、高危侵袭性B细胞淋巴瘤疗效及预后因素分析

**DOI:** 10.3760/cma.j.issn.0253-2727.2022.03.006

**Published:** 2022-03

**Authors:** 轶 王, 薇 刘, 文阳 黄, 瑞 吕, 健 李, 书会 邓, 伟薇 隋, 宏 刘, 婷玉 王, 树华 易, 慧敏 刘, 录贵 邱, 德慧 邹

**Affiliations:** 中国医学科学院北京协和医学院血液病医院（中国医学科学院血液学研究所），实验血液学国家重点实验室，国家血液系统疾病临床医学研究中心，细胞生态海河实验室，天津 300020 State Key Laboratory of Experimental Hematology, National Clinical Research Center for Blood Diseases, Haihe Laboratory of Cell Ecosystem, Institute of Hematology & Blood Diseases Hospital, Chinese Academy of Medical Sciences & Peking Union Medical College, Tianjin 300020, China

**Keywords:** 淋巴瘤，B细胞, 抗肿瘤联合化疗方案, 外周血干细胞移植, Lymphoma, B-cell, Antineoplastic combined chemotherapy protocols, Peripheral blood stem cell transplantation

## Abstract

**目的:**

探讨增强剂量免疫化疗联合自体外周血造血干细胞移植（ASCT）治疗初治、年轻、高危侵袭性B细胞淋巴瘤患者的疗效及预后因素。

**方法:**

回顾性分析2011年1月至2018年12月在中国医学科学院血液病医院应用增强剂量免疫化疗联合ASCT治疗的63例初治、年轻、高危侵袭性B细胞淋巴瘤患者的临床和生存资料。

**结果:**

63例患者的中位年龄为40（14～63）岁。诱导治疗方案包括R-DA-EP(D)OCH（52例）和R-HyperCVAD/R-MA（11例）。16例（25.4％）患者中期疗效评估为部分缓解，其中10例移植后获得完全缓解。中位随访50（8～112）个月，3年无进展生存（PFS）率和总生存（OS）率分别为（83.9±4.7）％和（90.4±3.7）％。单因素分析显示年龄调整的国际预后指数评分≥2分是影响OS的预后不良因素（*P*＝0.039），骨髓受累（BMI）是影响OS（*P*<0.001）和PFS（*P*＝0.001）的预后不良因素。多因素分析显示BMI是OS（*P*＝0.016）和PFS（*P*＝0.001）的唯一独立预后不良因素。

**结论:**

一线应用增强剂量免疫化疗联合ASCT治疗年轻、高危侵袭性B细胞淋巴瘤可获得良好的长期疗效，BMI为不良预后因素。

## Abstract (en)

**Objective:**

This study aimed to determine the efficacy of dose-enhanced immunochemotherapy followed by autologous peripheral blood stem cell transplantation（ASCT）in young patients with newly diagnosed high-risk aggressive B-cell lymphoma.

**Methods:**

A retrospective study was conducted to examine the clinical and survival data of young patients with high-risk aggressive B-cell lymphoma who received dose-enhanced immunochemotherapy and ASCT as first-line treatment between January 2011 and December 2018 in Blood Diseases Hospital.

**Results:**

A total of 63 patients were included in the study. The median age range was 40（14–63）years old. In terms of the induction therapy regimen, 52 cases received R-DA-EP（D）OCH, and the remaining 11 received R-HyperCVAD/R-MA. Sixteen（25.4％）patients achieved partial response in the mid-term efficacy assessment, and ten of them were evaluated as complete response after transplantation. The median follow-up was 50（8–112）months, and the 3-year progression-free survival（PFS）rate and overall survival（OS）rate were（83.9±4.7）％ and（90.4±3.7）％, respectively. Univariate analysis demonstrated that age-adjusted international prognostic index ≥2 scores was a negative prognostic factor for OS（*P*＝0.039）, and bone marrow involvement（BMI）was an adverse prognostic factor for OS（*P*<0.001）and PFS（*P*＝0.001）. However, multivariate analysis confirmed that BMI was the only independent negative predictor of OS（*P*＝0.016）and PFS（*P*＝0.001）.

**Conclusion:**

The use of dose-enhanced immunochemotherapy in combination with ASCT as first-line therapy in the treatment of young, high-risk aggressive B-cell lymphoma results in good long-term outcomes, and BMI remains an adverse prognostic factor.

侵袭性B细胞淋巴瘤是一类高度异质性肿瘤，高危患者采用R-CHOP方案治愈率不足50％[1]。多项研究对增强免疫化疗或免疫化疗联合新药、自体外周血造血干细胞移植（ASCT）巩固治疗在高危患者中的疗效及安全性进行了探索，其中一线ASCT的作用和价值存在争议，特别是在前期接受增强免疫化疗诱导的患者中。本研究回顾性分析了63例年轻、高危侵袭性B细胞淋巴瘤患者在我院应用增强剂量免疫化疗联合一线ASCT的疗效及预后因素，现将结果报道如下。

## 病例和方法

1. 病例：回顾性分析2011年1月至2018年12月在中国医学科学院血液病医院采用增强剂量免疫化疗联合一线ASCT治疗的63例年轻、高危侵袭性B细胞淋巴瘤患者的临床特征、疗效及生存情况。入选病例需满足以下条件：（1）年龄≤65岁。（2）具备以下至少1个高危因素：国际预后指数（IPI）评分≥3分或年龄调整的国际预后指数（aaIPI）评分≥2分、双/三打击淋巴瘤［DHL/THL，病理组织间期FISH示MYC重排阳性伴BCL2和（或）BCL6重排阳性］或进展期双表达淋巴瘤（DEL，Ann-Arbor分期Ⅲ～Ⅳ期伴病理组织免疫组化c-MYC阳性率≥40％且BCL2阳性率≥50％）、肿瘤细胞增殖指数（Ki-67）≥80％、中期疗效未达完全缓解（CR）。（3）病理类型包括：①弥漫大B细胞淋巴瘤（DLBCL）非特指型（DLBCL，NOS）；②转化的DLBCL（既往未治疗）；③高级别B细胞淋巴瘤（HGBCL）；④B细胞淋巴瘤，不可归类，其特征介于DLBCL和经典型霍奇金淋巴瘤之间；除外原发纵隔大B细胞淋巴瘤、原发性中枢神经系统DLBCL。疾病诊断标准参照世界卫生组织造血与淋巴组织肿瘤分型标准（2016版）[2]。

2. 治疗方案：增强剂量免疫化疗包括利妥昔单抗（375 mg/m^2^，第0天）联合Hyper-CVAD/MA（环磷酰胺+长春新碱/长春地辛+多柔比星/表柔比星+地塞米松与甲氨蝶呤+中剂量阿糖胞苷交替）或DA-EP（D）OCH（依托泊苷+泼尼松/地塞米松+长春新碱+环磷酰胺+多柔比星）方案。以上方案每21 d为1个疗程，具体用法及剂量调整见文献[3]–[4]。

造血干细胞动员化疗采用利妥昔单抗联合诱导化疗方案或大剂量依托泊苷方案（依托泊苷1.6～1.8 g/m^2^，第1天）。移植预处理方案包括R-BEAC/AEAC方案（利妥昔单抗+卡莫司汀/尼莫司汀+依托泊苷+阿糖胞苷+环磷酰胺）、R-GBC方案（利妥昔单抗+吉西他滨+白消安+环磷酰胺）和TBI（全身放疗）+R-EA/EC方案（利妥昔单抗+依托泊苷+环磷酰胺/阿糖胞苷）。

3. 疗效评价：治疗中期（3～4个疗程化疗后）或移植前及移植后2～3个月应用PET-CT评价疗效，其结果采用视觉判断法（IHP）或Deauville 5分法判读[5]–[6]。发病时有骨髓侵犯者在各疗效评价点均复查骨髓流式细胞术及病理免疫组化。疗效判断依据2014年修订的Lugano分期和疗效评价标准分为CR、部分缓解（PR）、疾病稳定和疾病进展[7]。

4. 随访：随访截止至2020年7月，中位随访时间为50（8～112）个月。随访资料来自住院病历、门诊病历及电话随访记录。随访期间死亡的病例根据病历记录或与患者家属电话联系确认。无进展生存（PFS）时间定义为自开始治疗至疾病复发/进展、任何原因导致的死亡或随访终止时间。总生存（OS）时间定义为从开始治疗至任何原因死亡或随访终止时间。

5. 统计学处理：采用SPSS 26.0软件进行统计学分析，采用Kaplan-Meier法计算PFS和OS并绘制生存曲线。单因素分析采用Log-rank检验，采用Cox回归分析进行多因素分析。双侧*P*<0.05为差异有统计学意义。

## 结果

1. 基线临床特征：63例患者的中位年龄40（14～63）岁，男28例（44.4％），女35例（55.6％），男女比例1∶1.25。病理类型为DLBCL，NOS的患者52例（82.5％），49例DLBCL可经Hans法行细胞起源分类，生发中心B细胞来源（GCB）和非生发中心B细胞来源（non-GCB）的患者分别为16例（32.7％）和33例（67.3％）；转化的DLBCL患者3例（4.8％），2例由滤泡性淋巴瘤转化，1例由边缘区淋巴瘤转化；病理类型介于DLBCL和霍奇金淋巴瘤之间的B细胞淋巴瘤患者2例（3.2％）；高级别B细胞淋巴瘤6例（9.5％）。52例（82.5％）患者Ann-Arbor分期为Ⅲ～Ⅳ期，11例（17.5％）患者Ann-Arbor分期为Ⅰ～Ⅱ期。59例患者可计算IPI或aaIPI评分，其年龄均不超过60岁。aaIPI评分为0～1分和2～3分的患者分别有19例（32.2％）和40例（67.8％）。39例（61.9％）患者初诊乳酸脱氢酶（LDH）升高，29例（46.8％）患者起病时伴B症状。

35例患者通过FISH检测了MYC、BCL2和（或）BCL-6重排情况，其中4例（11.4％）为DHL。39例患者具备免疫组化MYC和BCL2资料，符合DEL的23例（59.0％）患者中有3例也符合DHL。49例患者进行了Ki-67免疫组化检测，30例（61.2％）患者Ki-67≥80％。11例（18.6％）患者存在直径>7.5 cm的大包块。54例（85.7％）患者合并结外受累，最常见的结外受累部位为骨（35例，55.6％），其中18例（28.6％）患者经髂骨骨髓活检证实骨髓受累（BMI）。4例（6.3％）患者存在中枢神经系统侵犯。

2. 治疗情况：诱导治疗方面，52例（82.5％）患者采用了R-DA-EP（D）OCH方案，11例（17.5％）患者接受了R-HyperCVAD/R-MA方案。45例（71.4％）患者接受了鞘内注射或HD-MTX（大剂量甲氨蝶呤）进行中枢神经系统侵犯预防或治疗。29例（46.0％）和34例（54.0％）患者分别选用诱导化疗方案和大剂量依托泊苷作为动员方案。预处理选用R-BEAC/AEAC方案、R-GBC方案和TBI+R-EA/EC方案的患者例数分别为37例（58.7％）、16例（25.4％）和10例（15.9％）。移植前所有患者中位接受了6（4～14）个疗程化疗，整个治疗过程中位应用利妥昔单抗8（3～13）次。10例（15.9％）患者因预处理方案接受了TBI。4例（6.3％）患者进行了局部放疗，1例为中枢神经系统受累行颅脑放疗，1例为获得CR后对既往大包块区域放疗，2例为对残留病灶进行放疗。

3. 疗效及生存：所有患者均获得PR及以上疗效，治疗中期、移植前和移植后的CR率分别为74.6％、84.1％和90.5％。中期PET-CT（iPET）阳性的16例患者中有7例更换二线免疫化疗方案，其余患者继续按原方案治疗。其中11例患者于移植前再次评价疗效，6例达到CR，ASCT前CR率为84.1％。18例BMI患者中，仅1例患者移植前骨髓流式细胞术微小残留病（MRD）检测阳性，其余患者经骨髓病理免疫组化或MRD检测证实获得骨髓缓解。移植后CR率则进一步提升至90.5％。

截至末次随访，中位PFS及OS时间均未达到。3年PFS率和OS率分别为（83.9±4.7）％和（90.4±3.7）％，预计5年PFS率和OS率分别为（79.1±5.5）％和（83.5±5.2）％。

4例DHL患者的最佳疗效均为CR，其中1例DHL患者于ASCT后获得最佳疗效。DHL患者的中位随访时间为43（22～71）个月，均未发生进展或死亡。

4. 预后因素：对可能影响预后的因素进行单因素Kaplan-Meier分析，BMI是OS（*P*<0.001）和PFS（*P*＝0.001）的预后不良因素（图1），aaIPI≥2分是OS（*P*＝0.039）的预后不良因素。LDH高于3倍正常上限、伴B症状、non-GCB、DHL/DEL、Ki-67≥80％、合并大包块、骨受累、中枢神经系统侵犯、中期疗效、ASCT预处理方案对PFS和OS均无明显影响（表1）。进一步多因素分析显示，BMI是OS（*P*＝0.016）和PFS（*P*＝0.001）的唯一独立预后不良因素。

**图1:**
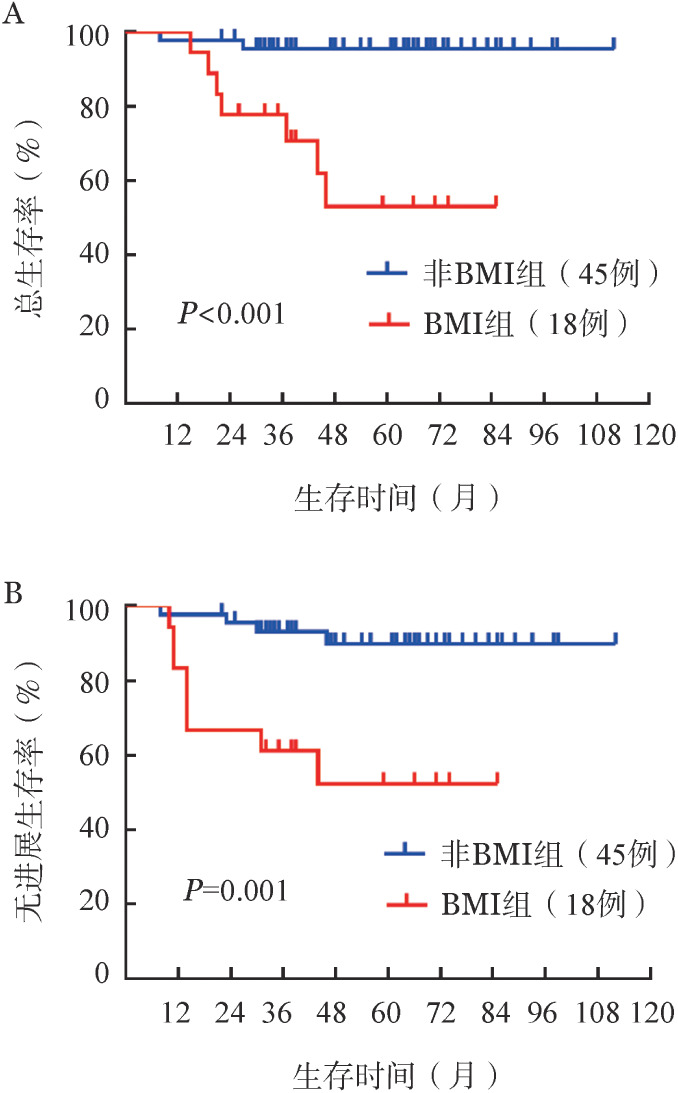
骨髓受累（BMI）组与非BMI组高危侵袭性B细胞淋巴瘤患者的总生存（A）和无进展生存（B）曲线

**表1:** 63例高危侵袭性B细胞淋巴瘤患者3年PFS和OS相关变量单因素分析结果

因素	例数	PFS			OS		
		3年PFS率（％，*x±s*）	*χ*^2^值	*P*值	3年OS率（％，*x±s*）	*χ*^2^值	*P*值
aaIPI			2.638	0.104		4.249	0.039
≥2分	40	79.8±6.4			87.4±5.3		
<2分	19	94.4±5.4			100.0		
LDH			1.546	0.214		1.162	0.281
≥3倍ULN	8	100.0			100.0		
<3倍ULN	50	83.9±5.2			90.0±4.2		
B症状			0.275	0.600		0.163	0.686
有	29	86.1±6.5			89.7±5.7		
无	33	81.6±6.8			90.7±5.1		
细胞来源			1.570	0.210		1.014	0.314
non-GCB	33	81.8±6.7			87.9±5.7		
GCB	16	93.8±6.1			93.8±6.1		
DHL/DEL			0.868	0.351		2.027	0.154
是	24	91.7±5.6			95.8±4.1		
否	17	88.2±7.8			94.1±5.7		
Ki-67指数			0.927	0.336		0.157	0.692
≥80％	30	89.7±5.6			100.0		
<80％	19	94.7±5.1			94.7±5.1		
大包块			2.737	0.098		2.089	0.148
有	11	100.0			100.0		
无	48	78.9±5.9			87.4±4.8		
骨受累			0.005	0.942		0.007	0.936
有	21	83.9±4.7			90.5±6.4		
无	42	85.7±7.6			90.3±4.6		
骨髓受累			11.622	0.001		13.166	<0.001
有	18	61.1±11.5			77.8±9.8		
无	45	93.2±3.8			95.4±3.1		
中枢神经系统侵犯			0.090	0.764		0.448	0.503
有	4	75.0±21.7			75.0±21.7		
无	59	84.5±4.8			91.4±3.7		
中期疗效			0.309	0.578		0.266	0.606
完全缓解	47	84.9±5.3			93.6±3.6		
部分缓解	16	81.3±9.8			80.8±10.0		
预处理方案			1.848	0.397		3.844	0.146
R-BEAC/AEAC方案	37	83.8±6.1			89.2±5.1		
R-GBC方案	16	93.8±6.1			100.0		
TBI+R-EA/EC方案	10	70.0±14.5			80.0±12.6		

注：PFS：无进展生存；OS：总生存；aaIPI：年龄调整的国际预后指数；LDH：乳酸脱氢酶；ULN：正常值上限；non-GCB：非生发中心B细胞来源；GCB：生发中心B细胞来源；DHL/DEL：双打击淋巴瘤/双表达淋巴瘤；R-BEAC/AEAC：利妥昔单抗+卡莫司汀/尼莫司汀+依托泊苷+阿糖胞苷+环磷酰胺；R-GBC：利妥昔单抗+吉西他滨+白消安+环磷酰胺；TBI+R-EA/EC：全身放疗+利妥昔单抗+依托泊苷+环磷酰胺/阿糖胞苷

## 讨论

以DLBCL为主的侵袭性B细胞淋巴瘤是一组潜在可治愈的恶性血液肿瘤，但其临床表现及生物学行为均呈现高度异质性[8]–[10]，高危患者接受传统免疫化疗后多在短期内复发进展，预后差[1]。DLCL04试验[11]、Alliance/GALGB 50303试验[12]及Cortelazzo等[13]的研究证实，密度和（或）剂量增强的R-CHOP-14方案、R-DA-EPOCH和R-MegaCHOP-14方案等均可改善高危患者的疗效及生存。但一线ASCT的地位目前仍存在争议，尤其是剂量/密度增强的免疫化疗诱导后的ASCT。

既往多项研究对一线ASCT的价值进行了探索。SWOG9704研究显示ASCT可明显提高接受CHOP21±R方案治疗的初治患者的2年PFS率（69％对55％，*P*＝0.005），并延长IPI≥4分患者的OS时间[14]。DSHNHL 2002-1临床试验结果则提示密度增强的免疫化疗（R-CHOEP14）疗效好，但进一步增强剂量（R-MegaCHOEP14）并联合一线ASCT不能延长生存期[15]。在随后的两项研究中，一线ASCT联合增强免疫化疗的无失败生存或无事件生存优势并未转化为PFS或OS获益，因此NCCN指南等目前不常规推荐高危患者行一线ASCT巩固治疗[11],[13]。然而，以上研究均仅通过IPI或aaIPI评分定义高危，未纳入具有其他生物学或缓解动力学高危因素的患者，可能遗漏潜在适应证。且上述研究的诱导治疗方案强度大（尤其是移植组），部分患者无法顺利完成ASCT或出现严重治疗相关不良反应，降低了移植组整体的疗效及预后。此外，ASCT是非移植组患者挽救治疗的重要组成部分，非一线应用ASCT带来的OS获益可能是上述研究未获得显著生存差异的主要原因。但考虑到我国国情及淋巴瘤诊疗现状，在一线治疗中即加深缓解深度、降低复发率或许更具可行性，且能一定程度上降低高危患者的整体治疗费用。

除IPI评分、aaIPI评分外，本研究涵盖更多目前认知的预后不良因素，如DHL/DEL、高Ki-67指数和中期疗效未达CR，通过扩充高危定义增加入组患者的代表性[16]–[18]。免疫化疗则选用剂量增强的R-DA-EP（D）OCH或R-HyperCVAD/R-MA方案，并适时予粒细胞集落刺激因子等支持治疗，患者整体表现出良好的耐受性。行ASCT前后的CR率分别为84.1％和90.5％，提示增强免疫化疗序贯ASCT仍能进一步加深疾病缓解深度。长期随访显示，患者3年PFS率和OS率分别为（83.9±4.7）％和（90.4±3.7）％，预计5年PFS率和OS率则分别为（79.1±5.5）％和（83.5±5.2）％，生存率较上述研究明显提高，表明高危侵袭性B细胞淋巴瘤经增强剂量免疫化疗序贯一线ASCT治疗可获得良好的长期疗效。

本研究的预后分析显示，增强免疫化疗联合一线ASCT治疗可初步克服DHL/DEL、中期疗效未达CR等高危因素的不良影响，与已有研究结论一致[3],[19]–[22]。而我们既往的研究证实BMI且骨髓活检肿瘤细胞比例>10％是高危DLBCL患者PFS（*P*＝0.024）和中枢神经系统复发进展（*P*＝0.005）的独立危险因素[4],[23]。2014年提出的NCCN-IPI评分也将BMI、骨受累区分，强调BMI对DLBCL预后的影响[24]。本研究中，BMI仍为PFS和OS的唯一独立预后不良因素，提示即使增强诱导治疗强度并联合一线ASCT，仍无法完全克服BMI的不良影响。对于这部分患者，未来仍需探索更有效的治疗手段，如联合新药、嵌合抗原受体T细胞（CAR-T细胞）治疗、异基因造血干细胞移植等。值得注意的是，aaIPI≥2分在单因素分析中是OS的预后不良因素（*P*＝0.039），但在多因素分析中未显示差异有统计学意义，主要考虑伴BMI患者Ann-Arbor分期均为Ⅳ期，与aaIPI≥2分患者存在较多重合，两因素间存在相互影响。

本研究也存在一定的局限性。首先，这是一项单中心回顾性研究，受纳入病例来源及例数的影响，部分研究结论可能存在一定偏倚；另外，患者经诱导治疗达PR及以上疗效才进入后续ASCT治疗，不能反映少部分原发难治高危患者的疗效及生存率。需要在前瞻性研究中进一步基于更完善的精确诊断、预后分层结合敏感的治疗反应评价，探索对年轻、高危侵袭性B细胞淋巴瘤的优化治疗策略。

综上，我们的研究显示增强剂量免疫化疗联合一线ASCT治疗年轻、高危侵袭性B细胞淋巴瘤获得了良好的长期疗效，但BMI仍为独立预后不良因素，有待探索更优的治疗选择。
